# Work accident effect on the use of psychotropic drugs: the case of benzodiazepines

**DOI:** 10.1186/s13561-023-00464-5

**Published:** 2023-10-23

**Authors:** Thomas Barnay, François-Olivier Baudot

**Affiliations:** 1 ERUDITE, Université Paris-Est Créteil, 61 Avenue du Général de Gaulle, Créteil, 94010 France; 2 Direction de la Stratégie, des Études et des Statistiques, Caisse Nationale de l’Assurance Maladie, 50 Avenue du Professeur André Lemierre, Paris, 75986 France

**Keywords:** Work, Accident, Occupational accident, Drug, Benzodiazepine, Overuse, Overconsumption, SNDS, France

## Abstract

**Background:**

A work accident constitutes a shock to health, likely to alter mental states and affect the use of psychotropic drugs. We focus on the use of benzodiazepines, which are a class of drugs commonly used to treat anxiety and insomnia. Prolonged use can lead to dependence. Our objective is to determine the extent to which work accidents lead to benzodiazepine use and overuse (i.e. exceedance of medical guidelines).

**Method:**

We use a two-step selection model (the Heckman method) based on data from the French National Health Data System (*Système National des Données de Santé*, SNDS). Our study sample includes all general plan members who experienced a single work accident in 2016 (and not since 2007). This sample includes 350,000 individuals in the work accident group and more than 1.1 million people randomly drawn from the population without work accidents from 2007 to 2017 (the non-work accident group).

**Results:**

The occurrence of a work accident leads to an increase in benzodiazepine use and overuse the following year. The selection model shows a clear influence of the accident on the use probability (+ 39%), but a very slight impact on the risk of overuse among users (+ 1.7%), once considered the selection effect. The effect on overuse risk is higher for more severe accidents and among women.

**Conclusion:**

The increase in the risk of benzodiazepine overuse is due to an increase in the likelihood of using benzodiazepines after a work accident that leads to overuse, rather than an increase in likelihood of overuse among people who use benzodiazepines. Results call for targeting the first-time prescription to limit the risk of overuse after a work accident.

**Supplementary Information:**

The online version contains supplementary material available at 10.1186/s13561-023-00464-5.

## Background

In 2016, in France, 715,130 work accidents (WAs) (including 13% of commuting accidents) were recorded, resulting in 768 deaths, more than 40,000 permanent disabilities, and €5.6 billion of daily allowances [1]. In total, nearly 4% of employees insured under the general scheme were victims of WAs during that year (authors’ calculation based on SNDS data). These WAs have countless effects on health, related to the great variety of jobs, people, and circumstances. In particular, they may impair mental health and lead to depression, anxiety, sleep disorders, and post-traumatic stress disorders (PTSD) [2, 3]. The degradation of mental health is related to the degree of impairment [4, 5]. Moreover, some studies show persistence during years after the accident of these psychiatric symptoms [6, 7], including road accidents (which represent a particular case of occupational accidents) [8, 9].

These psychiatric symptoms can be treated with various medications. Among them, benzodiazepines (BZDs) are especially useful because they are indicated to treat anxiety and trouble sleeping. Although controversial, they are also used to treat PTSD or along with antidepressant drugs at the beginning of antidepressant treatment [10, 11]. Like other drugs, they have several adverse effects, like psychomotor impairment, resulting in motor vehicle accidents and falls [12]. Moreover, extended use is associated with a loss of treatment efficacy and can lead to dependence, with an increased risk of withdrawal symptoms at standstill [13–15], so it is a source of inefficiency of treatment. To limit this risk, the French National Authority for Health (HAS) recommends limiting the duration of prescriptions to four weeks for hypnotic BZDs and twelve weeks for anxiolytic BZDs [16, 17]. Yet, in 2014 in France, 14% of new BZD users consumed the drug beyond the recommended treatment time [18].

The frequency of WAs raises the question of their impact on BZD overuse. Because a WA may lead to deteriorating mental health, BZDs are indicated in short-term treatment and so likely to be prescribed. However, prolonged use could result in inefficiency of treatment and be counterproductive.

Sex and age appear as the main drivers of BZD use. Sixty-five percent of consumers in France in 2015 were women and usage increased with age [18]. The use of psychotropic drugs (including BZDs) was also related to the socioeconomic group, with executives and higher intellectual professions consuming less than those in intermediate professions, employees, and workers [19]. Nearly 90% of BZD prescriptions are made by general practitioners (GPs), and 82% of new BZDs treatments are initiated by GPs [18].

Concerning overuse, the risk factors vary according to the criteria retained (duration of treatment or several BZDs taken simultaneously). In studies using duration of treatment as criteria of BZD overuse, the main drivers are advanced age, comorbidities (in particular history of depression and sleep disorders), other drugs used (in particular antidepressants and psychotropic drugs), and socio-professional characteristics (low educational level, low household income, and not having a job) [20–23]. The impact of sex on BZD overuse is not consensual: in some studies, BZD overuse is associated with being a man [20, 21], but sometimes the association is not significant [22].

In addition, the role of prescriber characteristics on inappropriate prescriptions (not focused on psychotropic drugs) is not well established in the literature. Prescriptions by psychiatrists or multiple prescribers seem to be risk factors [23–25]. Dhalla et al. [24] show an effect of prescriber characteristics (other predictors were male sex, age 50 and over, in general practice, and practice in a rural area), while others do not [26–28].

Work characteristics may also impact BZD consumption. In most cases, employment protects health status [29]; however, it can sometimes be pathogenic [30–32]. There are no studies that specifically address the impact of work on BZD use, but many studies stressed the impact of work characteristics on psychotropic drug use, in the case of psychosocial work factors [33, 34] and low job satisfaction [20]. Other job-related factors play a role in psychotropic drug use such as organizational downsizing [35, 36] and mass layoffs (effect on remaining employees) [37]. However, as far as we know, there is no published study on the impact of WA on psychotropic drug use.

Our objective is to measure the change in consumption of BZDs following the occurrence of a WA. The question is twofold: because WAs affect mental health, they could indirectly result in higher psychotropic drug consumption, and, in the case of BZD, extended use could be especially harmful because of the risk of dependence and this process feeds on itself.

To answer this question, we use the French National Health Data System (SNDS), which is an administrative database that contains all information related to reimbursed care in France, for the whole population. We distinguish between the use and overuse of BZDs, overuse being defined as non-compliance with the maximum recommended duration of treatment. We proceed in two steps: first, we use a two-step selection model to estimate the probability of BZD use and overuse after a WA; second, we focus on the population with WA to estimate the impact of severity of accident on BZD use and overuse.

Considering both the incidence of WAs and the important use of BZDs in France (and associated adverse events), the consequences of WAs on BZD use is a major question that has not been answered, to our knowledge. We provide here the first consistent analysis of the impact of a WA on BZD use and overuse. Moreover, previous studies analyzing risk factors of BZD overuse did not consider the selection effect in overuse (the need to be a user before overusing) and therefore confused factors associated with use and overuse. Our study shows that WAs increase the risk of BZD use and overuse. However, the increase in overuse comes from an increase in the likelihood of using after the WA, not from a large increase in the likelihood of overusing among those who use. Nevertheless, among people with WAs, the longer the subsequent work interruption, the higher the probability of overusing BZDs.

## Methods

### Data

The data come from the French National Health Data System (SNDS), which is a nationwide administrative database produced and managed by statutory health insurance (CNAM). It contains all information relating to reimbursements made by the CNAM (outpatient care, hospitalization, and cash benefits) [38]. It also contains data related to WAs and occupational diseases (ATMP in French), and it is used by the eponymous branch to reimburse insured persons, adjust firm pricing, and prevent occupational risks.

The information system makes it possible to know the exact dates of drug dispensation. It contains the following information on patients: year of birth, sex, department of residence, recipient of universal complementary health insurance (called CMU-C), and registration in a long-term disease scheme (called ALD), which allows exemption from user fees for care relating to registered diseases. It also contains information about prescribers (such as medical specialty, sex, and age). The exact dates and circumstances of WAs are also known. Finally, ATMP data were available from 2006 to 2017. Information on non-ATMP care was available from 2015 to 2017. Statistical analyses were performed using SAS 9.4 on Red Hat Enterprise Linux Server 7.4.

Using SNDS data, the CNAM produces the *Healthcare Expenditures and Conditions Mapping*, which allows patients to be classified into 58 nonexclusive groups according to their health status and treatments. This classification is based on reimbursements specific to some diseases, medical diagnosis during hospitalization, and registration as an ALD if applicable, according to the methodology described by Rachas et al. [39]. We use the year 2015.

### Scope of the study

The study covers the entire French population insured under the general scheme of the welfare system, i.e., employees in the private sector (except farmers) and civil servants. It covers the period from 2015 to 2017.

The inclusion criteria are as follows: having at least one treatment reimbursed by the general scheme in 2015 and 2016, and being between 18 and 65 years of age in 2016 (selection of a working-age population). We exclude persons who died before January 1, 2018, with an occupational injury (WA or professional disease) from 2007 to 2015 or in 2017 and with more than one occupational injury in 2016.

Our study population is composed of both people who have had a single WA in 2016 (and none other since 2007) (WA group) and people who have not had any WA between 2007 and 2017 (non-WA group). We consider only recognized WAs, and relapses are not considered. The selection of the period 2007–2017 for the non-WA group avoids a disruptive effect related to another damage and therefore allows identification of a “pure” effect of WAs occurring in 2016. Finally, because of the volume of data, we make a random selection of one-twentieth of the population that did not experience a WA from 2007 to 2017.

### Definition of use and overuse

In 2016, 19 different BZDs (including two related drugs: zolpidem and zopiclone) were marketed in pharmacies in France. We include all of these in this study. All BZDs have close effects and differ by their kinetics [40], short-acting BZDs are rather used as hypnotics and long-acting are rather used as anxiolytics, but there are possible substitutions. The homogeneity of the drug class in terms of effects justifies considering them all together.

Purchase dates are calculated around the WA date, occurring in 2016. To be able to calculate use in the same way for people with and without WAs, the WAs dates of the WA group are randomly distributed to the non-WA group. A month is assimilated to a 30-day period since prescriptions are often monthly and benzodiazepine boxes have a capacity of 30 tablets. A year corresponds to 12 times 30-day periods.

At least one BZD purchase defines use. Overuse corresponds to at least four months with BZDs issued in five consecutive months. According to the recommendations, the maximum duration of treatment with BZDs is twelve weeks of treatment for anxiolytics and four weeks for hypnotics (HAS, 2017, 2018). Overuse, therefore, corresponds to noncompliance with the recommended treatment times for anxiolytic BZDs. We apply the same rule to hypnotics for reasons of simplicity and homogeneity. We assume that at least four months with at least one dispensation for five consecutive months can characterize at least twelve weeks of continuous consumption, considering the variability that there may be in dispensation dates.

### Econometric strategy

We estimate the causal effect of the occurrence of a WA on BZD use and overuse. Estimating the overuse using a logit or probit may lead to biased results. Indeed, overuse can only exist among people who consume BZDs, and if the factors associated with use and overuse differ, there is a potential selection bias. We use a two-step selection method to consider this bias [41, 42]. It consists of estimating via a probit model the probability of using BZDs (BZDuse^*^_i_) in the first step (selection equation):

1 $$\textrm{P}\left({\textrm{BZDuse}}_{\textrm{i}}^{\ast }=1|{\textrm{X}}^{\prime}\right)=\Phi \left({\textrm{X}}_{\textrm{i}}^{\prime}\upbeta \right)$$

With Φ() the probability density function of a normal distribution and

$${X}_{i}^{\prime}\beta = {\beta }_{0}+{WA}_{i}^{\prime}{\beta }_{1}+{age}_{i}^{\prime}{\beta }_{2}+{sex}_{i}^{\prime}{\beta }_{3}+{urbanArea}_{i}^{\prime}{\beta }_{4}+{Xinsurance}_{i}^{\prime}{\beta }_{5}+{XpastBZD}_{i}^{\prime}{\beta }_{6}+{Xhealt{h}_{i}}^{\prime}{\beta }_{7}+{u}_{i}$$

Then, we estimate the probability of overusing BZDs (BZDoveruse^*^_i_) in the population using BZDs after the WA date in the second step (outcome equation):

2 $$\textrm{P}\left({\textrm{BZDoveruse}}_{\textrm{i}}^{\ast }=1|{\textrm{Z}}^{\prime}, {\textrm{BZDuse}}_{\textrm{i}}^{\ast } =1\right)=\Phi \left({\textrm{Z}}_{\textrm{i}}^{\prime}\updelta \right)$$

With

$${Z}_{i}^{\prime}\delta = {\delta }_{0}+{WA}_{i}^{\prime}{\delta }_{1}+{age}_{i}^{\prime}{\delta }_{2}+{sex}_{i}^{\prime}{\delta }_{3}+{urbanArea}_{i}^{\prime}{\delta }_{4}+{Zinsurance}_{i}^{\prime}{\delta }_{5}+{ZpastBZD}_{i}^{\prime}{\delta }_{6}+{Zhealt{h}_{i}}^{\prime}{\delta }_{7}+{Zprescriber}_{i}^{\prime}{\delta }_{8}+\uprho {\uplambda }_{\mathrm{i}}+{\varepsilon }_{i}$$

Where

$${BZDoveruse}_{i}={BZDoveruse}_{i}^{*}\mathrm{\ if\ }{BZDuse}_{i}^{*}>0$$

$${BZDoveruse}_{i}=0\mathrm{\ if\ }{BZDuse}_{i}^{*}\le 0$$

In the first step, we explain the BZD use in the year following the WA by a dummy variable for WA in 2016 (*WA’*); the age in 2016 (*age’*); the sex (*sex’*, ref. = male); a variable describing the urban area in 2016 by labor market size [43] (*urbanArea’*, 10 modalities); a vector of insurance-related variable (*Xinsurance’*_i_): CMU-C in 2015 (0/1), aid for the payment of supplementary health insurance (called ACS) in 2015 (0/1), and disabled adults’ allowance (called AAH) in 2015 (0/1), which are mean-tested benefits and therefore provide information on income levels; a vector (*XpastBZD’*_*i*_) of BZD use during the year preceding the WA (four dummies for at least one BZD use each quarter of the year); and a vector of health-related variables (*Xhealth’*_i_) including 15 dummies of health variables in 2015 from the *Healthcare Expenditures and Conditions Mapping*: cancers, cardioneurovascular diseases, treatment of vascular risk, inflammatory or rare disease or HIV/AIDS, neurological or degenerative diseases, psychiatric disorders, chronic end-stage renal disease, chronic respiratory disease diabetes, liver or pancreas disease, addictions, other long-term diseases, maternity, antidepressant dispensation, and dispensation of neuroleptics; *u*_*i*_ is the error term.

In the second step, variables explaining BZD overuse in the year following the WA are close to the first step. The past BZD use (*ZpastBZD’*_*i*_) includes a dummy of BZD overuse in the year preceding the WA in addition to the four dummies for BZD use in the quarters of the year. The vector of insurance (*Zinsurance’*_*i*_) is similar to *Xinsurance’*_*i*_. Health (*Zhealth’*_*i*_) variables are similar to *Xhealth’*_*i*_, except for *inflammatory or rare disease or HIV/AIDS (0/1)* which is absent. *Zprescriber’*_*i*_ is a vector of characteristics of the prescriber of the first BZD after the WA: medical specialty (GP, psychiatrist, another specialist, non-physician, multiple BZD prescribers the same day, or missing information), age (three modalities), and sex. Other variables are unchanged. Finally, ρλ_i_ is the inverse of the Mill ratio and ε_i_ is the error term.

The choice of variables should control for most of the determinants of BZD use and overuse found in the literature: demographics, economic disadvantage, chronic illnesses and use of other psychotropic drugs, and prescriber characteristics. The past use of BZDs is also supposed to be a strong predictor of current use, and it controls for reverse causality, as BZD use may increase the risk of WAs [44].

The identification of the model relies on the assumption of nonlinearity of the Mill inverse ratio, but it may be nonrobust due to collinearity [45]. It is recommended to use an identification variable, which would be a good predictor of *BZDuse* and would not be used in Eq. 2. Identifying such a variable (that is a variable influencing BZD use without influencing BZD overuse) is not an easy task, because the two outcomes are closely related, and previous studies did not consider the selection effect in overusing estimation. We used the variable *inflammatory or rare disease or HIV/AIDS (0/1)* as an identification variable because it is positively associated with BZD use, but not with BZD overuse in the estimation without an identification variable. These results are presented in “Results” section.

As robustness checks, we provide in the Additional file 1 estimations without an identification variable and using sex as an identification variable. Indeed, while being a woman is positively associated with BZD use, overuse is not significantly associated with sex according to some studies. Conversely, other studies show that substance use disorders are more common among men [46]. Results are available in Additional file 1. Nevertheless, the results of the main estimation show a significant effect of being a woman in the two steps (positive on BZD use and negative on BZD overuse), which goes against the use of this variable as an identification variable. Its non-significance in previous studies on BZD overuse may come from the non-consideration of selection and the opposite direction of sex effect on use and overuse.

The test for the correlation of the error terms shows that the null hypothesis must be rejected for all the specifications, which means that there is indeed a selection bias that must be corrected and that the ‘naïve’ model is biased. We provide in Table I in the Additional file 1 a ‘naïve’ probit estimation: an estimate of the probability of overuse for the population that consumed the year following the WA (which corresponds to the second step of the Heckman model).

### Descriptive statistics

Table 1 compares sociodemographic characteristics in WA and non-WA groups. The WA group is younger (78.5% are under 50 compared to 65.7% in the non-WA group), more often male (51% compared to 41%), and less disadvantaged. These differences refer to a selection bias. People with WA are people who worked at least once in 2016, while people without WA are people who used at least one treatment in 2015 and 2016 but whose employment status is unknown. This selection effect clearly appears in terms of health and healthcare expenditure heterogeneity. Thirty-one percent of the sample had at least one disease, with 34% in the non-WA group but only 23% in the WA group. Statistics relative to health status are shown in Table A in the Additional file 1. Whatever the disease, the WA group is healthier than the non-WA one. This group also has less frequent maternity leave and drug treatments. Differences in the typology of the municipality of residence are presented in Table B in the Additional file 1.

**Table 1:** Descriptive statistics

**Variables**	**WA group**	**Non-WA group**
*Sociodemographic variables in 2016*		
Average age	37.5 years	42.0 years
18–29	33.5%	22.9%
30–39	23.0%	21.1%
40–49	21.9%	21.8%
50–59	18.7%	21.6%
60–65	2.9%	12.7%
Male	51%	41%
CMU–C	9.4%	12.1%
ACS	3.8%	4.1%
AAH	0.9%	3.2%
*BZD consumption in the year preceding the WA*		
At least one BZD use	12.15%	15.08%
BZD overuse	2.89%	5.30%
*BZD consumption in the year following the WA (variation compare to the previous year)*		
At least one BZD use	16.79% (+ 38%)	14.93% (-1%)
BZD overuse calculated on 8 months	3.72% (+ 29%)	5.36% (+ 1%)
BZD overuse^a^	4.30%	5.92%
Observations	353,792	1,105,177

Source: SNDS

Field: People with WA in 2016 in France and randomly selected people without WA (*N* = 1,458,969)

Interpretation: In the study population, the average age of those who experienced a WA in 2016 is 37.5 years

Significance: All figures in this table are significantly different between both groups at the 0.1% threshold

^a^BZD overuse is not comparable between the year before and the year after the WA, because of the four months lagged required

We see in Table 1 statistics relative to BZD use and overuse. People with a WA in 2016 widely increased their use of BZDs during the following year (+ 38%) and overuse in a slightly smaller proportion (+ 29%, calculated over eight months after the WA to be comparable to the year before, because of the 4 months lagged required). In the group without WA, BZD use and overuse are higher; the slight decrease in use (-1%) refers to the downward trend in BZD consumption in France during the 2010s [18], but with a slight increase in overuse (+ 1%).

**Fig. 1:**
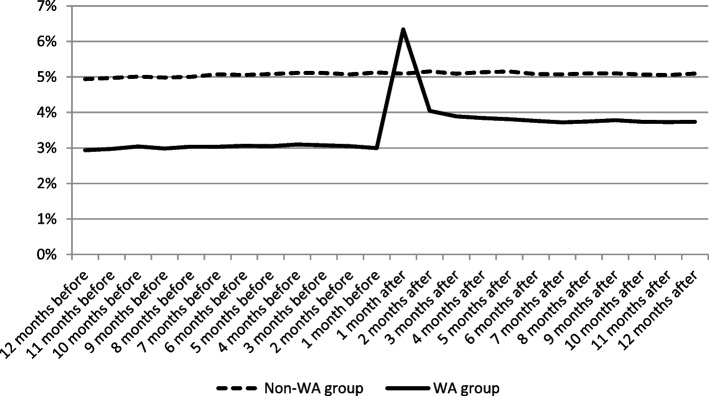
Percentage of monthly BZD users in both groups. Source: SNDS. Field: People with WA in 2016 in France and randomly selected people without WA (*N* = 1,458,969). Time refers to months before and after the date of the accident (in the WA group), occurring in 2016

Figure 1 shows that the proportion of BZD users in the non-WA group is stable during the two-year study period, and higher than the proportion of people with WA, before the WA occurrence. There is a very significant increase in BZD use in the month following the WA, and then it decreases but remains at a higher level than before the WA.

The majority of the population is made up of individuals who had no BZD dispensation: 85% in the non-WA group and 88% in the WA group during the year before the accident (85% and 83%, respectively, for the following year). For the others, most had a single dispensation: 5.6% of people in the non-WA group and 5.8% of people in the WA group the year before the accident (5.5% and 8%, respectively, for the year after). Among people in the WA group who had a single dispensation in the year following the accident, 27% had a single dispensation in the month following the accident.

During the year following the WA, in the population using a BZD at least one time, a GP prescribed the first BZD in 92% of cases in the WA group, vs. 87% in the non-WA group. After a WA, the ascertainment and declaration of the accident are usually done by a GP, which explains this difference if the GP prescribes the BZD during the same consultation. More statistics about prescribers of the first BZD after the WA are available in Additional file 1: Table C.

## Results

### Effect of the occurrence of a WA on BZD use and overuse

The results of the main estimation are presented in Table 2. The left-hand column shows the result of the selection equation, that is the estimation of BZD use. A WA occurrence led to an increase by 5.9 percentage points (pp) in the probability of using BZDs the following year, i.e., a 39.3% increase. This is consistent with the statistic of BZD use shown in Table 1. The right-hand column shows results regarding BZD overuse; the WA led to an increase of 0.006 pp, i.e., 1.67%, among the population who used it. This is far smaller than the increase in BZD overuse presented in Table 1.

**Table 2:** Estimated impact of WA on the use and overuse of BZDs the following year

**Variables**	**Effect of the WA on BZD use**	**Effect of the WA on BZD overuse**
Population	1,458,969	224,371
Mean of the dependent variable	15%^a^	36%^b^
Marginal effect of WA (pp)	0.059^***^	0.006^***^
Mean effect on the dependent variable	+ 39.3%^***^	+ 1.67%^***^
Sociodemographic controls	Yes	Yes
Municipality of residence	Yes	Yes
Past BZD use	Yes	Yes
Health status	Yes	Yes
Prescriber characteristics	NA	Yes
ρ (correlation coefficient of error terms)	NA	0.29^***^

Source: SNDS

Field: People with WA in 2016 in France and randomly selected people without WA (*N* = 1,458,969)

Interpretation: Suffering from a WA in 2016 increases the probability of having had at least one BZD use the following year by 39.3%

*NA* not applicable

^***^p < 0.001

^a^Percentage of people using BZDs the year following WA in the whole population

^b^Percentage of people overusing BZDs the year following WA among the population who used it

The correlation coefficient of error terms (ρ) is significant at the 0.1% threshold, confirming the selection effect in BZD overuse and leading to reject the probit without selection correction (results are presented in Table I in the Additional file 1).

BZD use (see Table E in the Additional file 1) increases with past BZD use, age, being a woman, markers of social disadvantage (CMU-C, ACS, and AAH), and bad health (except diabetes). The association with motherhood is negative. The typology of the municipality of residence shows, when significant, that middle hubs and isolated municipalities are associated with lower use compared to large hubs.

Regarding overuse (see Table F in the Additional file 1), we see a positive effect of age, being a man, markers of social disadvantage, living on the outskirts of large hubs and small municipalities, past BZD use (in particular overuse in the preceding year), and suffering from a chronic illness. The probability of overuse increases when a psychiatrist or multiple prescribers on the same day make the first BZD prescription after the WA. Other specialists are associated with less overuse; the age and sex of the prescriber are insignificant.

We identify a possible dose–response relationship of WA to BZD use. To do this, we repeat the analyses on the population who were victims of a WA in 2016, using the prescribed duration of sick leave following the accident as a proxy for the severity of the accident. The variable is divided into quartiles (7, 15, and 45 days). We also add the salary as a control variable in the model, recalculated from the amount of the sickness benefits (which is capped). The sample is therefore composed of individuals who had a WA followed by at least one day off work. The results are summarized in Table 3. Full results are available in Tables G and H in the Additional file 1.

**Table 3:** Impact of sick leave duration on BZD use and overuse

Sick leave duration	Population	Coefficients (SE)
*Estimation of use*		
≤ 7 days	250,791	Ref
> 7 days and ≤ 15 days		0.045^***^ (0.0094)
> 15 days and ≤ 45 days		0.1073^***^ (0.0091)
> 45 days		0.3794^***^ (0.0088)
*Estimation of overuse*		
≤ 7 days	46,280	Ref
> 7 days and ≤ 15 days		-0.0031 (0.0278)
> 15 days and ≤ 45 days		0.0276 (0.0266)
> 45 days		0.3945^***^ (0.0287)

Source: SNDS

Field: WA group (without missing information about sick leave) (*N* = 250,791)

^***^p < 0.001

The longer the duration of sick leave, the greater the probability of consuming a BZD in the year following the WA. Similarly, if the effect of the shortest stops is not significant, the longest stops (beyond 45 days) lead to an increased risk of overuse in the year following the WA.

### Robustness checks

The robustness checks aim to account for the selection effect related to employment status and measure and methodological issues. The results are summarized in Table 4, the first column being the result of the main estimation, as a reference.

**Table 4:** Robustness checks, results of the outcome equation (estimation of BZD overuse)

	**Main model**	**Check 1**	**Check 2**	**Check 3**	**Check 4**	**Check 5**	**Check 6**	**Check 7**
Coefficient of WA variable (SE)	0.1099^***^ (0.0099)	0.1107^***^ (0.0099)	0.1176^***^ (0.0097)	0.0806^***^ (0.018)	0.0543 (0.0349)	0.1011^***^ (0.0107)	0.082^***^ (0.0114)	0.1074^***^ (0.0097)
Identification variable	Inflammatory or rare disease or HIV/AIDS	**None**	**Sex**	Inflammatory or rare disease or HIV/AIDS	Inflammatory or rare disease or HIV/AIDS	Inflammatory or rare disease or HIV/AIDS	Inflammatory or rare disease or HIV/AIDS	**None**
Population	Selected population	Selected population	Selected population	**Population with sickness benefits**	**Matched population**	Selected population	Selected population	Selected population
Overuse variable	4 out of 5 months with BZDs	4 out of 5 months with BZDs	4 out of 5 months with BZDs	4 out of 5 months with BZDs	4 out of 5 months with BZDs	**5 out of 6 months with BZDs**	**6 out of 7 months with BZDs**	4 out of 5 months with BZDs
Health controls	15 health-related variables	15 health-related variables	15 health-related variables	15 health-related variables	15 health-related variables	15 health-related variables	15 health-related variables	**Decile of health expenditure**
Observations	224,371	224,371	224,371	50,476	70,512	224,371	224,371	224,371

Source: SNDS

^***^p < 0.001

The first two checks are related to the identification variable. The use of the variable *inflammatory or rare disease or HIV/AIDS (0/1)* seems to us the most relevant choice, given its positive association with BZD use but not overuse. Nevertheless, we report the results of two others specifications (see Table J in the Additional file 1): without an identification variable (in this case, the identification relies on the assumption of nonlinearity of the Mill inverse ratio), and using sex as the identification variable. The results are identical to those of the main estimation.

Check 3 is the most important. We focus on the subpopulation of sickness benefits recipients in 2015. As already mentioned and checked using descriptive statistics, we are faced with a serious selection effect related to employment status, the WA group being employed, and the employment status of the non-WA group being unknown (combining employees and non-employees). There is no available variable on employment status in the database. The use of this subpopulation reduces this selection bias; indeed, it is composed of people with employment in 2015. We assume that this is still the case in 2016. We include in the model the number of days compensated in 2016 and the average daily amount, which is a proxy for salary (for descriptive statistics, see Table D in the Additional file 1). The results are almost identical to those obtained for the selected population. This test greatly increases our confidence in the results, because the employment status does not appear to bias the results. The full results are available in Tables K (for the first step) and L (for the second step) in the Additional file 1. The decile of sickness benefits (i.e. wage) is negatively associated with both BZD use and overuse; the decile of sick leave duration in 2015 is only associated with the probability of BZD overuse.

Large differences exist in BZD consumption between both groups. To account accurately for BZD dispensation in the year before WA, we conduct check 4: an exact matching (with replacement). The matching variables are as follows: sociodemographic variables (age, sex, CMU-C, ACS, AAH beneficiary in 2015); BZDs lagged variables (before a WA) (number of BZD dispensation each month (12 variables) and number of different BZDs dispensed each month (12 variables)); and the decile of reimbursable health expenses in 2015. Thanks to the large dataset, 333,347 cases are matched to 300,656 controls, i.e. a matching rate of 94%. The coefficient of the WA variable in the second step is close to the main estimation but non-significant. The slightly higher coefficient in the first step (see Table K in the Additional file 1) and the non-significant coefficient in the second step (see Table L in the Additional file 1) reinforce the finding following the main estimation: when the previous BZD use is accurately controlled in the model, the WA occurrence increases the probability to use BZDs, but not the probability to overuse BZDs among users.

We test two new definitions of overuse in checks 5 and 6: at least five months with at least one BZD dispensation in six consecutive months, and at least six months with BZD dispensation in seven consecutive months, respectively. These two new overuse variables are used as variables explained in the second step of the model. They are also used as control variables in these estimations (for overuse in the year before WA). Results are close to estimates with the previous overuse variable and are available in Table M in the Additional file 1.

Finally, in the baseline model, health status is defined based on 15 diseases. These cover a limited number of individuals since 69% suffer from no disease. Therefore, we test variants using the decile of reimbursable expenditure in 2015 in check 7 (the reimbursable expenditure corresponds to the total amount of care provided and not the amount actually reimbursed by the statutory health insurance). Once again, results are similar to those of the main estimation. Full results of the first and second steps are available in Tables N and O in the Additional file 1.

The results are reinforced by robustness checks. None of the results deviate significantly from the main estimate, particularly for the population with sickness benefits in 2015. This check strongly reduces the possibility that uncontrolled occupational status is a source of bias. In the main estimation, the occurrence of WA increases the BZD overuse by 0.006 pp, which is very small. The nonsignificance of the coefficient in check 4 indicates an even smaller (or null) effect on BZD overuse when past BZD use is properly controlled.

### Heterogeneity

In order to check for heterogeneity, we stratify our sample by sex. The results of estimations are summarized in Table 5. Full results are available in Tables P and Q in the Additional file 1. The occurrence of the WA appears to have a slightly higher effect on benzodiazepine use and overuse in the following year for women.

**Table 5:** Estimation of BZD use and overuse, stratified by sex

**Population**	**Observations**	**Coefficients (SE)**
*Estimation of BZD use*		
Men	634,786	0.2933^***^ (0.0052)
Women	824,183	0.3669^***^ (0.0044)
*Estimation of BZD overuse*		
Men	79,129	0.0986^***^ (0.0159)
Women	145,242	0.1181^***^ (0.013)

Source: SNDS

Field: People with WA in 2016 in France and randomly selected people without WA (*N* = 1,458,969)

^***^p < 0.001

## Discussion

### Discussion of results

The contribution of the two-step selection model is to distinguish between determinants of BZD use and overuse by considering the selection effect in BZD overuse. To our knowledge, this has not been done before in the literature. The occurrence of a WA, which is a shock to health (mental health in particular), increases the probability of BZD use and overuse, as observed in statistics (see Table 1 and Fig. 1). The selection model shows that the increase in BZD overuse comes from the increase in BZD use (+ 39% after the WA), which translates into overuse, and slightly from an increased risk of overuse among users (+ 1.7%), once corrected for selection bias. It means that experiencing a WA will increase the risk of overusing BZDs mainly by increasing the share of people with BZDs prescribed and not the propensity to overuse BZDs after starting to use them. Previous studies on factors associated with BZD overuse were based on people using BZD [21, 23], thus related to a specific and selected population of people already using BZDs, or comparing people with continuous use to the study population without controlling for the selection effect [20, 22]. The only study we found on the effect of an accident (in this case, a road traffic accident) compares only pre- and post-accident use [47].

Estimates of control variables are consistent with the literature: higher with increasing age, for women and disadvantaged people [18, 19]. Motherhood decreases the probability of BZD use (consistent with the recommendations for use during pregnancy). We see a particularly strong association with psychiatric chronic diseases and antidepressant treatment, which refers to comorbidities and the frequent association between antidepressants and BZDs. The typology of the municipality of residence shows, when significant, that middle hubs and isolated municipalities are associated with lower use compared to large hubs, which is consistent with previous literature that shows more use in urban areas [48].

Regarding overuse, being a woman has a protective effect, consistent with findings in the literature on the overuse of BZDs and other substances [21, 46, 49]. The association between insurance variables and BZD overuse shows an increased risk for economically disadvantaged people, in particular for AAH, which also refers to disability. The positive effect of the prescriber variable may come from more severe diseases in the case of psychiatrists and misuse in the case of multiple prescribers. This result is consistent with previous literature on factors associated with long-term prescription of BZD [23].

Among the population with WA (employed), the risk of both using and overusing BZDs increases with the duration of sick leave, i.e., with the severity of the accident (see Table 3). This result is consistent with the literature showing the harmful effect of occupational accidents on psychological health [2].

BZDs are prescription drugs, and their use refers both to the patients’ health demand and the prescribers’ behavior. After years of high consumption in France, public authorities have taken measures to reduce it. Successfully, since BZD consumption has decreased overall since the early 2000s [18]. Prescribing physicians are encouraged to limit the duration of BZD treatments because a portion of their pay depends on the share of their patients overusing BZDs [50]. Nevertheless, we see that in the WA group, the BZD use translates into overuse for 36% of people in the year following the WA (see Table 2).

These results have possible implications for public health policies. Results show that public authorities should target primary prescribing to limit the risks associated with BZD overuse after a WA. After removing the effect on BZD use, the risk of overusing BZDs after a WA increases very slightly, except for WAs resulting in a work interruption of more than 45 days. After a WA, the prescribers (mainly GPs) should question the need for BZDs, knowing the associated risk of overuse. Once prescribed, the focus should be on the more impaired workers to prevent the adverse effects of BZDs from adding to the consequences of the WA. Moreover, as previously shown in the literature, having multiple prescribers increases the risk of exceeding recommendations, so, wherever possible, prescribers should work together to avoid multiple prescriptions.

### Limitations

Some limitations come from the information system. We do not know if reimbursed drugs have been used. Regardless of whether the likelihood of unused medicine is significant for a single-box dispensation, we think it is very low for multiple deliveries. Socioeconomic variables are very scarce in the SNDS, which does not provide employment status and profession. That could bias the results if the profession is correlated with both employment and BZD use. Nevertheless, the closeness of results for the population with sickness benefits in 2015 addresses this objection even if the sample is small. This makes it possible to focus on the employed population in 2015 and to control for recalculated income, which is a strong proxy for the socio-professional category. Moreover, in estimations using the severity of WAs, the population is limited to the employed population, and values of WA benefits are used as a proxy of socioeconomic status (because they are directly related to salary).

The choice of BZD overuse variable can be discussed. Since medical diagnoses are not included in our database, we used a proxy of BZD use for an excessive duration compared to recommendations. Our results are robust to different definitions of the overuse variable. We did not take into account simultaneous use, because of the small case number, or distinguished hypnotic and anxiolytic BZDs, for reasons of simplicity and because of the homogeneity of the BZD class.

The study proposes to analyze the impact of a WA. However, the methodology applied does not allow us to attribute the effects to the occupational nature of the accident. An alternative approach to achieve this objective could compare one population with WAs to another one that experiences accidents outside the workplace. This approach is not feasible in this study due to data constraints. Additionally, an interesting extension of the study could involve a long-term effects evaluation of WAs. Unfortunately, this extension was not viable in our current study due to the limited availability of three years of data.

## Conclusions

France is a big consumer of anxiolytic and hypnotic medications, and, in particular, BZDs. Maximum consumption durations are often exceeded, and the non-respect of guidelines can lead to adverse effects, including dependence. Extended use of BZDs is a source of treatment inefficiency and should be avoided regardless of the underlying disease. It is a major concern to identify the factors that could lead to BZD overuse. We provide the first estimation of a WA impact on the risk of BZD overuse.

A WA increases the risk of BZD use and overuse. The selection model makes it possible to show that the effect on BZD overuse comes mainly from an increase in the new BZD consumption and not from an increase in the risk of overusing among BZD users. The risk of overusing BZDs increased among the most impaired workers and women.

In order to limit BZD overuse after a WA, these results call for limiting the first-time BZD prescriptions to those patients for whom it seems most necessary. BZDs showed limited benefits in treating anxiety and insomnia, and it is crucial that their possible adverse effects do not add to the consequences of WAs on health.

### Supplementary Information

**Additional file 1: Table A.** Health status differences in the WA and the non-WA groups in 2015. **Table B.** Typology of the municipality of residence in the WA and the non-WA groups. **Table C.** Characteristics of prescribers of the first BZD after the WA. **Table D.** Descriptive statistics for the population that received sick benefits in 2015. **Table E.** Results of the selection equation (1) in the study population. **Table F.** Results of the outcome equation (2) in the study population. **Table G.** Results of the selection equation (1) with the duration of sick leave in the WA group. **Table H.** Results of the outcome equation (2) with the duration of sick leave in the WA group. **Table I.** Results of the probit equation among BZD users after the WA. **Table J.** Coefficients (SE) of the outcome equation (2) with other identification variables. **Table K.** Coefficients (SE) of the selection equation (1) in the population with sickness benefits and the matched population. **Table L.** Coefficients (SE) of the outcome equation (2) in the population with sickness benefits and the matched population. **Table M.** Coefficients (SE) of the outcome equation (2) with other overuse variables. **Table N.** Results of the selection equation (1) with other health control variables. **Table O.** Results of the outcome equation (2) with other health control variables. **Table P.** Coefficients (SE) of the selection equation (1) by sex. **Table Q.** Coefficients (SE) of the outcome equation (2) by sex.

## Data Availability

The study was carried out on data from the SNDS database by François-Olivier Baudot, who is employed by the French National Health Insurance Fund (CNAM). According to the low (n°2016–41; JORF 2016), permanent access to the SNDS is granted to CNAM employees, under very strict conditions (presence of accreditations, secure access…).

## Abbreviations

AAH: *Allocation aux adultes handicapés* (Disabled adults’ allowance)
ACS: *Aide au paiement d’une complémentaire santé* (Aid for the payment of supplementary health insurance)
ANSM: *Agence nationale de sécurité du médicament et des produits de santé* (French national agency for the safety of medicines and health products)
ATMP: *Accidents du travail et maladies professionnelles* (Work accidents and professional diseases)
BZD: Benzodiazepines
CMU-C: *Couverture maladie universelle complémentaire* (Universal complementary health coverage)
CNAM: *Caisse nationale de l’assurance maladie* (French statutory health insurance)
HAS: *Haute autorité de santé* (French national authority for health)
PTSD: Post-traumatic stress disorder
SNDS: *Système national des données de santé* (French national health data system)
WA: Work accident
