# Supplementary material for: Work accident effect on the use of psychotropic drugs: the case of benzodiazepines
Source: Health Econ Rev. 2023 Oct 23;13:48. doi: 10.1186/s13561-023-00464-5 (PMC10594863; doi:10.1186/s13561-023-00464-5)
Supplement: Supplementary file 1 — Additional file 1: Table A. Health status differences in the WA and the non-WA groups in 2015. Table B. Typology of the municipality of residence in the WA and the non-WA groups. Table C. Characteristics of prescribers of the first BZD after the WA. Table D. Descriptive statistics for the population that received sick benefits in 2015. Table E. Results of the selection equation (1) in the study population. Table F. Results of the outcome equation (2) in the study population. Table G. Results of the selection equation (1) with the duration of sick leave in the WA group. Table H. Results of the outcome equation (2) with the duration of sick leave in the WA group. Table I. Results of the probit equation among BZD users after the WA. Table J. Coefficients (SE) of the outcome equation (2) with other identification variables. Table K. Coefficients (SE) of the selection equation (1) in the population with sickness benefits and the matched population. Table L. Coefficients (SE) of the outcome equation (2) in the population with sickness benefits and the matched population. Table M. Coefficients (SE) of the outcome equation (2) with other overuse variables. Table N. Results of the selection equation (1) with other health control variables. Table O. Results of the outcome equation (2) with other health control variables. Table P. Coefficients (SE) of the selection equation (1) by sex. Table Q. Coefficients (SE) of the outcome equation (2) by sex. [file 13561_2023_464_MOESM1_ESM.pdf]

Additional file

Additional statistics

Table A: Health status differences in the WA and the non-WA groups in 2015

Variables	WA group	Non-WA group
Vascular risk treatments (excluding pathologies)	6.81%	10.67%
Psychotropic treatments (excluding pathologies)§	6.09%	8.33%
Antidepressant, Lithium, Depakote, and Depamide treatments (excluding pathologies)	3.61%	4.77%
Chronic respiratory diseases	3.47%	4.17%
Maternity (with or without pathologies)	2.45%	4.35%
Psychiatric illnesses	2.28%	4.39%
Diabetes	2.24%	4.08%
Cardioneurovascular diseases	1.66%	3.24%
Inflammatory or rare diseases or HIV or AIDS	1.44%	1.98%
Cancers	1.33%	2.68%
Others long-lasting diseases	1.17%	1.93%
Neurologic or degenerative diseases	0.69%	1.38%
Liver or pancreas diseases	0.58%	0.88%
Addictive disorders	0.51%	0.75%
Neuroleptic treatments	0.26%	0.49%
End-stage renal diseases	0.04%	0.13%
Observations	353,792	1,105,177

Source: SNDS

Field: People with a WA in 2016 in France and randomly selected people without a WA (N = 1,458,969)

Reading guide: In the study population, 6.81% of people with a WA in 2016 are treated for a vascular risk in 2015.

Note: All figures in this table are statistically different between the WA and the non-WA groups at the 0.1% threshold.

§: Not used in the model because this variable includes BZD treatments.

Table B: Typology of the municipality of residence in the WA and the non-WA groups

Typology of the municipality of residence	WA group	Non-WA group
Municipality belonging to a large hub (10,000 or more jobs)	57.8%	61.69%
Municipality belonging to the outskirts of a large hub	18.62%	17.98%
Multipolarized municipality of large urban areas	5%	4.68%
Municipality belonging to a middle hub (5,000 to less than 10,000 jobs)	2.56%	2.72%
Municipality belonging to the outskirts of a middle hub	0.49%	0.47%
Municipality belonging to a small hub (from 1,500 to less than 5,000 jobs)	3.2%	3.04%
Municipality belonging to the outskirts of a small hub	0.22%	0.21%
Other multipolarized municipality	4.61%	4.21%
Isolated municipality outside a hub influence	3.25%	3.28%
Missing or inconsistent	4.25%	1.71%
Observations	353,792	1,105,177

Source: SNDS

Field: People with a WA in 2016 in France and randomly selected people without a WA (N = 1,458,969)

Reading guide: In the study population, 57.8% of people in the WA group are living in a municipality belonging to a large hub.

Table C: Characteristics of prescribers of the first BZD after the WA

	Non-WA group	WA group
Prescriber position		
General practitioner	61.62%	65.51%
Psychiatrist	6.64%	3.33%
Non-physician	0.40%	0.24%
Other medical specialties	2.37%	2.35%
Multiple BZD prescribers on the same day	0.15%	0.13%
Missing information	28.82%	28.44%
Prescriber sex		
Man	41.94%	42.61%
Woman	17.04%	17.83%
Missing information	41.01%	39.56%
Prescriber age		
Below 40	5.96%	7.20%
40-59	31.58%	33.38%
60+	21.45%	19.86%
Missing information	41.01%	39.56%

Source: SNDS.

Field: People using at least once a BZD after the WA date (N = 224,371) in the study population.

Reading guide: Among those who used at least once a BZD after the WA date, the first BZD was prescribed by a GP for 61.62% of people in the non-WA group.

Table D: Descriptive statistics for the population that received sick benefits in 2015

Variables	WA group	Non-WA group
Mean age in 2016	39 years	40 years
% Male	47%	40%
CMU-C in 2015	5.6%	4.5%
Average total expenditure repayable in 2015	€3,189	€5,090
At least one BZD use in the year preceding the WA	19.89%	20.87%
At least one BZD use the year following the WA	22.69%	18.38%
Average daily amount of sick leaves in 2015	€32	€39
Average sick leaves duration in 2015 (days)	34 days	52 days
Observations	97,354	154,448

Source: SNDS.

Scope: Population having received at least one daily allowance payment in 2015 for sickness (N = 251,802).

Interpretation: In this population, the average age in the WA group in 2016 was 39 years.

Significance: All figures in this table are statistically different between the WA group and the non-WA group at the 0.1% threshold.

Main results

Table E: Results of the selection equation (1) in the study population

	Coefficients (SE)	Marginal effects
WA	0.3342*** (0.0034)	0.0590
Demographic		
Age in 2016	0.0098*** (0.0001)	0.0016
Sex (ref. = male)	0.2019*** (0.0031)	0.0323
Insurance		
CMU-C	0.1087*** (0.0046)	0.0184
ACS	0.0406*** (0.0076)	0.0067
AAH	0.0843*** (0.0098)	0.0142
Typology of the municipality		
Large hub (10,000 or more jobs)	Ref.	Ref.
Outskirts of a large hub	-0.0057 (0.004)	-0.0009
Multipolarized municipality of large urban areas	-0.0042 (0.007)	-0.0007
Middle hub (5,000 to less than 10,000 jobs)	-0.025** (0.0092)	-0.0041
Outskirts of a middle hub	-0.0093 (0.0214)	-0.0013
Small hub (from 1,500 to less than 5,000 jobs)	0.003 (0.0085)	0.0006
Outskirts of a small hub	-0.0065 (0.0322)	-0.0011
Other multipolarized municipality	-0.0072 (0.0074)	-0.0012
Isolated municipality outside a hub influence	-0.0242** (0.0084)	-0.0038
Missing or inconsistent	-0.1781*** (0.0107)	-0.0263
Past BZD use		
BZD use in the last quarter of the year preceding the WA	1.1242*** (0.0058)	0.3032
BZD use in the third quarter of the year preceding the WA	0.7407*** (0.0061)	0.1728
BZD use in the second quarter of the year preceding the WA	0.5971*** (0.0063)	0.1306
BZD use in the first quarter of the year preceding the WA	0.589*** (0.0061)	0.1280
Health		
Cancers	0.0301** (0.0092)	0.0050
Cardioneurovascular diseases	0.0278** (0.0088)	0.0046
Vascular risk treatments (excluding pathologies)	0.0223*** (0.0051)	0.0036
Inflammatory or rare diseases or HIV or AIDS	0.0821*** (0.0104)	0.0141
Neurological or degenerative diseases	0.1327*** (0.0129)	0.0229
Psychiatric illnesses	0.4024*** (0.0086)	0.0804
Chronic end-stage renal disease	0.0342 (0.0438)	0.0034
Chronic respiratory diseases	0.1029*** (0.0072)	0.0175
Other long-term conditions	0.0548***	0.0092

	(0.011)	
Diabetes	-0.0558*** (0.0081)	-0.0088
Diseases of the liver or pancreas	0.0686*** (0.0158)	0.0117
Maternity (with or without pathologies)	-0.0215** (0.0081)	-0.0035
Addictive disorders	0.1027*** (0.0185)	0.0178
Antidepressant, lithium, Depakote, and Depamide treatments (excluding pathologies)	0.3726*** (0.0066)	0.0732
Neuroleptic treatments (excluding pathologies)	0.2155*** (0.0218)	0.0407

Source: SNDS.
Field: People with WA in 2016 in France and randomly selected people without WA (N = 1,458,969).
*Note: * $p < 0.05$, ** $p < 0.01$, *** $p < 0.001$.*

Table F: Results of the outcome equation (2) in the study population

	Coefficients (SE)	Marginal effects
WA	0.1099*** (0.0099)	0.0061
Demographic		
Age in 2016	0.0055*** (0.0004)	0.0002
Sex (ref. = male)	-0.03*** (0.0084)	-0.0016
Insurance		
CMU-C	0.1377*** (0.0106)	0.0080
ACS	0.0569*** (0.016)	0.0031
AAH	0.1947*** (0.0159)	0.0121
Typology of the municipality		
Large hub (10,000 or more jobs)	Ref.	Ref.
Outskirts of a large hub	0.0214* (0.0101)	0.0011
Multipolarized municipality of large urban areas	0.0516** (0.0173)	0.0028
Middle hub (5,000 to less than 10,000 jobs)	0.0411 (0.0221)	0.0022
Outskirts of a middle hub	0.081 (0.0528)	0.0045
Small hub (from 1,500 to less than 5,000 jobs)	0.0841*** (0.0199)	0.0047
Outskirts of a small hub	0.0285 (0.0805)	0.0015
Other multipolarized municipality	0.0769*** (0.0178)	0.0042
Isolated municipality outside a hub influence	0.0425* (0.02)	0.0023
Missing or inconsistent	0.0209 (0.0279)	0.0011
Past BZD use		
BZD use in the last quarter of the year preceding the WA	0.941*** (0.0163)	0.1053
BZD use in the third quarter of the year preceding the WA	0.3181*** (0.0139)	0.0220
BZD use in the second quarter of the year preceding the WA	0.1069*** (0.0132)	0.0062
BZD use in the first quarter of the year preceding the WA	0.1812*** (0.0123)	0.0111
BZD overuse in the previous year	1.3201*** (0.0128)	0.1771

Health		
Cancers	0.0528** (0.018)	0.0029
Cardioneurovascular diseases	0.1408*** (0.0164)	0.0084
Vascular risk treatments (excluding pathologies)	0.052*** (0.0102)	0.0028
Neurological or degenerative diseases	0.1839*** (0.0224)	0.0114
Psychiatric illnesses	0.4887*** (0.0134)	0.0384
Chronic end-stage renal disease	-0.0382 (0.0801)	-0.0019
Chronic respiratory diseases	0.1408*** (0.0138)	0.0083
Other long-term conditions	0.0442* (0.0205)	0.0024
Diabetes	0.1012*** (0.016)	0.0058
Diseases of the liver or pancreas	0.1286*** (0.0275)	0.0076
Maternity (with or without pathologies)	-0.0053 (0.0266)	-0.0003
Addictive disorders	0.0561* (0.0249)	0.0031
Antidepressant, lithium, Depakote, and Depamide treatments (excluding pathologies)	0.297*** (0.011)	0.0200
Neuroleptic treatments (excluding pathologies)	0.3154*** (0.0289)	0.0219
Prescriber position		
GP	Ref.	Ref.
Another specialist	-0.1336*** (0.0239)	-0.0146
Missing information	-0.5639*** (0.0137)	-0.0461
Multiple	0.2088* (0.0953)	0.0281
Non-physician	0.0734 (0.0586)	0.0091
Psychiatrist	0.1917*** (0.0154)	0.0255
Sex and age of the prescriber		
Missing information	0.0943*** (0.0179)	0.0046
Male prescriber	0.0154 (0.0102)	0.0007
Age < 40	Ref.	Ref.
Age 40-59	-0.0166 (0.0153)	-0.0009
Age > 59	0.0157 (0.0163)	0.0008
ρ (correlation coefficient of error terms)	0.2931*** (0.0266)	NA

Source: SNDS.
Field: People with WA in 2016 in France and randomly selected people without WA, using at least once a BZD after the WA date (N = 224,371).
*Note: * $p < 0.05$, ** $p < 0.01$, *** $p < 0.001$.*

Complementary analysis

Table G: Results of the selection equation (1) with the duration of sick leave in the WA group

	Coefficients (SE)
Duration of sick leave following the WA	
Sick leave ≤ 7 days	Ref.
Sick leave > 7 days and ≤ 15 days	0.045*** (0.0094)
Sick leave > 15 days and ≤ 45 days	0.1073*** (0.0091)
Sick leave > 45 days	0.3794*** (0.0088)
Sociodemographic	
Decile of daily wage	0.0077*** (0.0012)
Age in 2016	0.0074*** (0.0003)
Sex (ref. = male)	0.3165*** (0.0067)
Insurance	
CMU-C	0.1173*** (0.0106)
ACS	0.0639*** (0.0157)
AAH	0.0603 (0.0326)
Typology of the municipality	
Large hub (10,000 or more jobs)	Ref.
Outskirts of a large hub	-0.0062 (0.0085)
Multipolarized municipality of large urban areas	-0.0086 (0.0148)
Middle hub (5,000 to less than 10,000 jobs)	-0.0247 (0.0202)
Outskirts of a middle hub	-0.003 (0.046)
Small hub (from 1,500 to less than 5,000 jobs)	-0.0454* (0.0184)
Outskirts of a small hub	0.1503* (0.0642)
Other multipolarized municipality	-0.0055 (0.0154)
Isolated municipality outside a hub influence	-0.0156 (0.0181)
Missing or inconsistent	-0.2069*** (0.022)
Past BZD use	
BZD use in the last quarter of the year preceding the WA	0.9439*** (0.0144)
BZD use in the third quarter of the year preceding the WA	0.6742*** (0.0147)
BZD use in the second quarter of the year preceding the WA	0.5413*** (0.015)
BZD use in the first quarter of the year preceding the WA	0.5189*** (0.0148)
Health	
Cancers	0.0089 (0.0271)
Cardioneurovascular diseases	-0.0166 (0.0244)
Vascular risk treatments (excluding pathologies)	-0.0074 (0.0127)
Inflammatory or rare diseases or HIV or AIDS	0.0191 (0.0259)
Neurological or degenerative diseases	0.1909***

	(0.0359)
Psychiatric illnesses	0.401*** (0.0235)
Chronic end-stage renal disease	-0.2134 (0.1671)
Chronic respiratory diseases	0.0655*** (0.0166)
Other long-term conditions	0.0237 (0.029)
Diabetes	-0.1071*** (0.0211)
Diseases of the liver or pancreas	0.0406 (0.0391)
Maternity (with or without pathologies)	0.0043 (0.0203)
Addictive disorders	0.0749 (0.0465)
Antidepressant, lithium, Depakote, and Depamide treatments (excluding pathologies)	0.3445*** (0.0167)
Neuroleptic treatments (excluding pathologies)	0.2377*** (0.061)

Source: SNDS.
Field: WA group (without missing information about sick leave) (N = 250,791).
*Note: * $p < 0.05$, ** $p < 0.01$, *** $p < 0.001$.*

Table H: Results of the outcome equation (2) with the duration of sick leave in the WA group

	**Coefficients (SE)**
Duration of sick leave following the WA	
Sick leave ≤ 7 days	Ref.
Sick leave > 7 days and ≤ 15 days	-0.0031 (0.0278)
Sick leave > 15 days and ≤ 45 days	0.0276 (0.0266)
Sick leave > 45 days	0.3945*** (0.0287)
Sociodemographic variables	
Decile of daily wage	-0.0107*** (0.0032)
Age in 2016	0.0068*** (0.0009)
Sex (ref. = male)	-0.0914*** (0.0234)
Insurance	
CMU-C	0.0661* (0.0268)
ACS	0.0071 (0.0398)
AAH	0.1895** (0.0617)
Typology of the municipality	
Large hub (10,000 or more jobs)	Ref.
Outskirts of a large hub	-0.0015 (0.0227)
Multipolarized municipality of large urban areas	0.0357 (0.0388)
Middle hub (5,000 to less than 10,000 jobs)	-0.0394 (0.054)
Outskirts of a middle hub	0.138 (0.1184)
Small hub (from 1,500 to less than 5,000 jobs)	0.0939* (0.0454)
Outskirts of a small hub	0.0124 (0.1601)
Other multipolarized municipality	-0.0151

	(0.0406)
Isolated municipality outside a hub influence	0.0072<br>(0.047)
Missing or inconsistent	0.1166<br>(0.0663)
Past BZD use	
BZD use in the last quarter of the year preceding the WA	0.7307***<br>(0.0473)
BZD use in the third quarter of the year preceding the WA	0.195***<br>(0.0378)
BZD use in the second quarter of the year preceding the WA	0.0629<br>(0.034)
BZD use in the first quarter of the year preceding the WA	0.1089***<br>(0.0318)
BZD overuse in the previous year	1.3188***<br>(0.0333)
Health	
Cancers	-0.005<br>(0.0564)
Cardioneurovascular diseases	0.0319<br>(0.0504)
Vascular risk treatments (excluding pathologies)	0.0697**<br>(0.0265)
Neurological or degenerative diseases	0.1955**<br>(0.0717)
Psychiatric illnesses	0.3549***<br>(0.0399)
Chronic end-stage renal disease	-0.3081<br>(0.3368)
Chronic respiratory diseases	0.116***<br>(0.0349)
Other long-term conditions	0.0146<br>(0.0619)
Diabetes	0.0722<br>(0.0464)
Diseases of the liver or pancreas	0.0146<br>(0.0807)
Maternity (with or without pathologies)	0.0506<br>(0.0601)
Addictive disorders	0.0576<br>(0.0699)
Antidepressant, lithium, Depakote, and Depamide treatments (excluding pathologies)	0.257***<br>(0.0292)
Neuroleptic treatments (excluding pathologies)	0.4016***<br>(0.0844)
Prescriber position	
GP	Ref.
Another specialist	-0.1645**<br>(0.0554)
Missing information	-0.4009***<br>(0.0313)
Multiple	0.3071<br>(0.1887)
Non-physician	0.1411<br>(0.1672)
Psychiatrist	0.2729***<br>(0.0425)
Sex and age of the prescriber	
Missing information	0.0175<br>(0.0399)
Male prescriber	-0.0214<br>(0.0232)
Age < 40	Ref.
Age 40-59	0.0158<br>(0.0333)
Age > 59	0.0982**<br>(0.036)

ρ (correlation coefficient of error terms)	-0.0154<br>(0.0733)

Source: SNDS.
Field: WA group (without missing information about sick leave), people using BZD at least once after the WA date (N = 46,280)
*Note: * $p < 0.05$, ** $p < 0.01$, *** $p < 0.001$.*

Robustness checks

Results of the probit

Table I: Results of the probit equation among BZD users after the WA

	**Coefficients (SE)**
WA	0.06***<br>(0.009)
Demographic	
Age in 2016	0.0039***<br>(0.0004)
Sex (ref. = male)	-0.0623***<br>(0.008)
Insurance	
CMU-C	0.1206***<br>(0.0108)
ACS	0.0532**<br>(0.0162)
AAH	0.186***<br>(0.0161)
Typology of the municipality	
Large hub (10,000 or more jobs)	Ref.
Outskirts of a large hub	0.0233*<br>(0.0103)
Multipolarized municipality of large urban areas	0.0548**<br>(0.0177)
Middle hub (5,000 to less than 10,000 jobs)	0.0461*<br>(0.0225)
Outskirts of a middle hub	0.1033<br>(0.0539)
Small hub (from 1,500 to less than 5,000 jobs)	0.087***<br>(0.0203)
Outskirts of a small hub	0.0394<br>(0.0821)
Other multipolarized municipality	0.0821***<br>(0.0181)
Isolated municipality outside a hub influence	0.0473*<br>(0.0204)
Missing or inconsistent	0.0513<br>(0.0285)
Past BZD use	
BZD use in the last quarter of the year preceding the WA	0.7675***<br>(0.0094)
BZD use in the third quarter of the year preceding the WA	0.2084***<br>(0.0106)
BZD use in the second quarter of the year preceding the WA	0.0185<br>(0.011)
BZD use in the first quarter of the year preceding the WA	0.1017***<br>(0.0104)
BZD overuse in the previous year	1.3663***<br>(0.0117)
Health	
Cancers	0.0494**<br>(0.0182)
Cardioneurovascular diseases	0.1384***<br>(0.0167)

Vascular risk treatments (excluding pathologies)	0.0513***<br>(0.0104)
Inflammatory or rare diseases or HIV or AIDS	0.0223<br>(0.0216)
Neurological or degenerative diseases	0.1654***<br>(0.0226)
Psychiatric illnesses	0.4408***<br>(0.013)
Chronic end-stage renal disease	-0.0517<br>(0.0814)
Chronic respiratory diseases	0.1316***<br>(0.014)
Other long-term conditions	0.0352<br>(0.0208)
Diabetes	0.1115***<br>(0.0162)
Diseases of the liver or pancreas	0.1194***<br>(0.028)
Maternity (with or without pathologies)	-0.002<br>(0.0275)
Addictive disorders	0.0369<br>(0.025)
Antidepressant, lithium, Depakote, and Depamide treatments (excluding pathologies)	0.2491***<br>(0.0104)
Neuroleptic treatments (excluding pathologies)	0.2972***<br>(0.029)
Prescriber position	
GP	Ref.
Another specialist	-0.1316***<br>(0.0244)
Missing information	-0.5779***<br>(0.0138)
Multiple	0.2515**<br>(0.0975)
Non-physician	0.0891<br>(0.06)
Psychiatrist	0.1901***<br>(0.0156)
Sex and age of the prescriber	
Missing information	0.0931***<br>(0.0183)
Male prescriber	0.016<br>(0.0105)
Age < 40	Ref.
Age 40-59	-0.0172<br>(0.0156)
Age > 59	0.0158<br>(0.0167)

Source: SNDS.
Field: People with WA in 2016 in France and randomly selected people without WA, using at least once a BZD after the WA date (N = 224,371).
*Note: * $p < 0.05$, ** $p < 0.01$, *** $p < 0.001$.*

Other identification variables

Table J: Coefficients (SE) of the outcome equation (2) with other identification variables

	Without an identification variable	Sex as the identification variable
WA	0.1107*** (0.0099)	0.1176*** (0.0097)
Demographic		
Age in 2016	0.0055*** (0.0004)	0.0057*** (0.0004)
Sex (ref. = male)	-0.0296*** (0.0084)	NA
Insurance		
CMU-C	0.1388*** (0.0106)	0.14*** (0.0106)
ACS	0.0567*** (0.016)	0.0544*** (0.0159)
AAH	0.1945*** (0.0159)	0.1999*** (0.0158)
Typology of the municipality		
Large hub (10,000 or more jobs)	Ref.	Ref.
Outskirts of a large hub	0.0215* (0.0101)	0.0212* (0.01)
Multipolarized municipality of large urban areas	0.052** (0.0173)	0.0538** (0.0172)
Middle hub (5,000 to less than 10,000 jobs)	0.0401 (0.0221)	0.0353 (0.022)
Outskirts of a middle hub	0.0815 (0.0528)	0.076 (0.0526)
Small hub (from 1,500 to less than 5,000 jobs)	0.0847*** (0.0199)	0.0825*** (0.0198)
Outskirts of a small hub	0.0267 (0.0805)	0.023 (0.0802)
Other multipolarized municipality	0.0775*** (0.0178)	0.0796*** (0.0177)
Isolated municipality outside a hub influence	0.0427* (0.02)	0.0396* (0.0199)
Missing or inconsistent	0.0218 (0.0279)	0.0219 (0.0277)
Past BZD use		
BZD use in the last quarter of the year preceding the WA	0.9424*** (0.0163)	0.9592*** (0.0154)
BZD use in the third quarter of the year preceding the WA	0.3198*** (0.0139)	0.3299*** (0.0135)
BZD use in the second quarter of the year preceding the WA	0.1066*** (0.0132)	0.117*** (0.0129)
BZD use in the first quarter of the year preceding the WA	0.1813*** (0.0123)	0.1889*** (0.012)
BZD overuse in the previous year	1.3192*** (0.0128)	1.3128*** (0.0128)
Health		
Cancers	0.0524** (0.018)	0.0498** (0.0179)
Cardioneurovascular diseases	0.1401*** (0.0164)	0.1473*** (0.0162)
Vascular risk treatments (excluding pathologies)	0.0522*** (0.0102)	0.0528*** (0.0102)
Inflammatory or rare diseases or HIV or AIDS	0.0348 (0.0213)	0.0359 (0.0213)
Neurological or degenerative diseases	0.1834*** (0.0224)	0.1837*** (0.0223)
Psychiatric illnesses	0.4903*** (0.0134)	0.4935*** (0.0132)
Chronic end-stage renal disease	-0.0387	-0.0362

	(0.0801)	(0.0798)
Chronic respiratory diseases	0.1395***	0.1429***
	(0.0138)	(0.0138)
Other long-term conditions	0.0428*	0.0423*
	(0.0205)	(0.0205)
Diabetes	0.1008***	0.1006***
	(0.016)	(0.0159)
Diseases of the liver or pancreas	0.1266***	0.1325***
	(0.0276)	(0.0274)
Maternity (with or without pathologies)	-0.005	-0.0135
	(0.0266)	(0.0262)
Addictive disorders	0.0568*	0.0631*
	(0.0249)	(0.0247)
Antidepressant, lithium, Depakote, and Depamide treatments (excluding pathologies)	0.2972***	0.2985***
	(0.011)	(0.0109)
Neuroleptic treatments (excluding pathologies)	0.3166***	0.3275***
	(0.0289)	(0.0287)
Prescriber position		
GP	Ref.	Ref.
Another specialist	-0.1329***	-0.1284***
	(0.0239)	(0.0237)
Missing information	-0.5627***	-0.5613***
	(0.0137)	(0.0137)
Multiple	0.1978*	0.1726
	(0.0951)	(0.0944)
Non-physician	0.0707	0.067
	(0.0586)	(0.0582)
Psychiatrist	0.1921***	0.191***
	(0.0154)	(0.0153)
Sex and age of the prescriber		
Missing information	0.0936***	0.0967***
	(0.0179)	(0.0178)
Male prescriber	0.0156	0.0169
	(0.0102)	(0.0102)
Age < 40	Ref.	Ref.
Age 40-59	-0.0167	-0.0165
	(0.0153)	(0.0152)
Age > 59	0.0158	0.016
	(0.0163)	(0.0162)
ρ (correlation coefficient of error terms)	0.2954***	0.3291***
	(0.0267)	(0.0255)

Source: SNDS.
Field: People with WA in 2016 in France and randomly selected people without WA, using at least once a BZD after the WA date (N = 224,371).
*Note: * $p < 0.05$, ** $p < 0.01$, *** $p < 0.001$.*

Other populations

Table K: Coefficients (SE) of the selection equation (1) in the population with sickness benefits and the matched population

	Population with sickness benefits in 2015	Matched population
WA	0.2685*** (0.0067)	0.3376*** (0.0044)
Decile of sickness benefits	-0.0062*** (0.0013)	NA
Decile of sick leave duration in 2015	0.0023 (0.0013)	NA
Demographic		
Age in 2016	0.0088*** (0.0003)	0.0104*** (0.0002)
Sex (ref. = male)	0.2385*** (0.0073)	0.2481*** (0.0045)
Insurance		
CMU-C	0.1011*** (0.015)	0.1354*** (0.0074)
ACS	0.0676*** (0.0189)	0.083*** (0.0116)
AAH	0.0156 (0.0295)	-0.0289 (0.0249)
Typology of the municipality		
Large hub (10,000 or more jobs)	Ref.	Ref.
Outskirts of a large hub	-0.0026 (0.0083)	-0.0061 (0.0058)
Multipolarized municipality of large urban areas	-0.0128 (0.0147)	-0.0099 (0.0102)
Middle hub (5,000 to less than 10,000 jobs)	-0.0221 (0.021)	-0.0547*** (0.014)
Outskirts of a middle hub	0.0625 (0.0437)	-0.0162 (0.0313)
Small hub (from 1,500 to less than 5,000 jobs)	0.018 (0.0188)	-0.0264* (0.0128)
Outskirts of a small hub	0.0246 (0.0669)	0.0338 (0.0456)
Other multipolarized municipality	-0.0092 (0.0153)	-0.0156 (0.0107)
Isolated municipality outside a hub influence	-0.0402* (0.0188)	-0.0261* (0.0125)
Missing or inconsistent	-0.0437 (0.0255)	-0.267*** (0.0147)
Past BZD use		
BZD use in the last quarter of the year preceding the WA	1.0045*** (0.0117)	0.9814*** (0.0119)
BZD use in the third quarter of the year preceding the WA	0.6439*** (0.0118)	0.7521*** (0.0125)
BZD use in the second quarter of the year preceding the WA	0.5026*** (0.0118)	0.6752*** (0.0127)
BZD use in the first quarter of the year preceding the WA	0.4525*** (0.0115)	0.6394*** (0.0123)
Health		
Cancers	-0.0146 (0.0181)	0.0057 (0.017)
Cardioneurovascular diseases	-0.0093 (0.0187)	-0.0064 (0.0164)
Vascular risk treatments (excluding pathologies)	0.0305** (0.0115)	0.0187* (0.0085)
Inflammatory or rare diseases or HIV or AIDS	0.0734*** (0.0206)	0.0657*** (0.017)
Neurological or degenerative diseases	0.1134*** (0.0302)	0.1901*** (0.0235)

Psychiatric illnesses	0.3757***<br>(0.0185)	0.4757***<br>(0.0161)
Chronic end-stage renal disease	0.0198<br>(0.0942)	-0.1119<br>(0.1008)
Chronic respiratory diseases	0.0642***<br>(0.0153)	0.101***<br>(0.0114)
Other long-term conditions	0.0442<br>(0.024)	0.0325<br>(0.019)
Diabetes	-0.0433*<br>(0.0194)	-0.0701***<br>(0.0146)
Diseases of the liver or pancreas	0.0528<br>(0.0323)	0.1067***<br>(0.0275)
Maternity (with or without pathologies)	-0.1689***<br>(0.0134)	-0.0345**<br>(0.0125)
Addictive disorders	0.1394***<br>(0.0393)	0.1095**<br>(0.034)
Antidepressant, lithium, Depakote, and Depamide treatments (excluding pathologies)	0.283***<br>(0.0127)	0.4748***<br>(0.0113)
Neuroleptic treatments (excluding pathologies)	0.2124***<br>(0.0508)	0.3109***<br>(0.0418)

Source: SNDS.
Field: Left: study population with sickness benefits (N = 251,802); Right: WA group and matched non-WA group (N = 666,694).
*Note: * $p < 0.05$, ** $p < 0.01$, *** $p < 0.001$.*

Table L: Coefficients (SE) of the outcome equation (2) in the population with sickness benefits and the matched population

	Population with sickness benefits in 2015	Matched population
WA	0.0806***<br>(0.018)	0.0543<br>(0.0349)
Decile of sickness benefits	-0.0166***<br>(0.0031)	NA
Decile of sick leave duration in 2015	0.0081**<br>(0.0029)	NA
Demographic		
Age in 2016	0.0069***<br>(0.0009)	0.0081***<br>(0.0013)
Sex (ref. = male)	-0.0271<br>(0.0196)	-0.0731**<br>(0.0275)
Insurance		
CMU-C	0.0582<br>(0.033)	0.1392***<br>(0.0278)
ACS	0.0238<br>(0.0421)	0.061<br>(0.0403)
AAH	0.1722**<br>(0.0525)	0.1525*<br>(0.0716)
Typology of the municipality		
Large hub (10,000 or more jobs)	Ref.	Ref.
Outskirts of a large hub	0.0046<br>(0.0197)	-0.0091<br>(0.02)
Multipolarized municipality of large urban areas	0.0651<br>(0.0337)	0.0882**<br>(0.0339)
Middle hub (5,000 to less than 10,000 jobs)	0.0319<br>(0.0475)	-0.0455<br>(0.0507)
Outskirts of a middle hub	0.0613<br>(0.1032)	0.0264<br>(0.1083)
Small hub (from 1,500 to less than 5,000 jobs)	0.1216**<br>(0.041)	0.0663<br>(0.0426)
Outskirts of a small hub	0.0749<br>(0.153)	-0.0642<br>(0.1596)
Other multipolarized municipality	0.0497<br>(0.0355)	0.0005<br>(0.0372)
Isolated municipality outside a hub influence	-0.0135	0.0753

	(0.0437)	(0.0411)
Missing or inconsistent	0.1003<br>(0.0585)	0.1854**<br>(0.0579)
Past BZD use		
BZD use in the last quarter of the year preceding the WA	0.944***<br>(0.0335)	0.4066***<br>(0.0896)
BZD use in the third quarter of the year preceding the WA	0.3198***<br>(0.0289)	-0.0637<br>(0.0671)
BZD use in the second quarter of the year preceding the WA	0.0972***<br>(0.027)	-0.0047<br>(0.0634)
BZD use in the first quarter of the year preceding the WA	0.1796***<br>(0.024)	-0.0149<br>(0.0603)
BZD overuse in the previous year	1.1641***<br>(0.0276)	2.4115***<br>(0.1254)
Health		
Cancers	0.0266<br>(0.0358)	0.0794<br>(0.0484)
Cardioneurovascular diseases	0.0951**<br>(0.0357)	-0.0112<br>(0.0494)
Vascular risk treatments (excluding pathologies)	0.0688**<br>(0.0219)	0.0536*<br>(0.0249)
Neurological or degenerative diseases	0.1963***<br>(0.0546)	0.164*<br>(0.0662)
Psychiatric illnesses	0.3933***<br>(0.0292)	0.4644***<br>(0.0595)
Chronic end-stage renal disease	-0.1146<br>(0.1771)	-0.6273<br>(0.5198)
Chronic respiratory diseases	0.1078***<br>(0.0295)	0.1162***<br>(0.0346)
Other long-term conditions	-0.027<br>(0.0463)	0.0057<br>(0.057)
Diabetes	0.0859*<br>(0.0393)	0.1263**<br>(0.0433)
Diseases of the liver or pancreas	-0.0361<br>(0.06)	0.0501<br>(0.0773)
Maternity (with or without pathologies)	-0.0467<br>(0.0404)	0.0425<br>(0.0469)
Addictive disorders	0.1034*<br>(0.0512)	0.0226<br>(0.0755)
Antidepressant, lithium, Depakote, and Depamide treatments (excluding pathologies)	0.2378***<br>(0.0219)	0.3238***<br>(0.051)
Neuroleptic treatments (excluding pathologies)	0.3146***<br>(0.0674)	0.4192***<br>(0.0917)
Prescriber position		
GP	Ref.	Ref.
Another specialist	-0.0864<br>(0.0501)	-0.1971***<br>(0.05)
Missing information	-0.5783***<br>(0.0294)	-0.4176***<br>(0.0266)
Multiple	0.2485<br>(0.1859)	0.8007***<br>(0.2066)
Non-physician	0.0353<br>(0.1453)	-0.027<br>(0.1328)
Psychiatrist	0.1623***<br>(0.0301)	0.419***<br>(0.0393)
Sex and age of the prescriber		
Missing information	0.17***<br>(0.0367)	0.1182**<br>(0.0369)
Male prescriber	0.0279<br>(0.0207)	-0.0387<br>(0.0217)
Age < 40	Ref.	Ref.
Age 40-59	0.025<br>(0.0305)	0.0165<br>(0.0315)
Age > 59	0.0495<br>(0.0328)	0.0988**<br>(0.0339)
ρ (correlation coefficient of error terms)	0.3117***<br>(0.067)	-0.1036<br>(0.1155)

Source: SNDS.
Field: Left: people using BZDs at least once among the study population with sickness benefits (N = 50,476); Right: people using BZDs at least once among the WA group and the matched non-WA group (N = 70,512).
*Note: * $p < 0.05$, ** $p < 0.01$, *** $p < 0.001$.*

Other overuse variables

Table M: Coefficients (SE) of the outcome equation (2) with other overuse variables

	Overuse: 5 out of 6 months with BZDs	Overuse: 6 out of 7 months with BZDs
WA	0.1011***<br>(0.0107)	0.082***<br>(0.0114)
Demographic		
Age in 2016	0.0046***<br>(0.0004)	0.0034***<br>(0.0005)
Sex (ref. = male)	-0.0512***<br>(0.0089)	-0.0556***<br>(0.0094)
Insurance		
CMU-C	0.1471***<br>(0.0113)	0.1391***<br>(0.0118)
ACS	0.0437**<br>(0.0166)	0.0606***<br>(0.0171)
AAH	0.2031***<br>(0.0161)	0.2023***<br>(0.0162)
Typology of the municipality		
Large hub (10,000 or more jobs)	Ref.	Ref.
Outskirts of a large hub	0.0125<br>(0.0109)	0.0207<br>(0.0115)
Multipolarized municipality of large urban areas	0.053**<br>(0.0186)	0.0669***<br>(0.0196)
Middle hub (5,000 to less than 10,000 jobs)	0.0373<br>(0.0235)	0.0524*<br>(0.0245)
Outskirts of a middle hub	0.1178*<br>(0.0568)	0.07<br>(0.0615)
Small hub (from 1,500 to less than 5,000 jobs)	0.075***<br>(0.0212)	0.1013***<br>(0.0221)
Outskirts of a small hub	0.0591<br>(0.0856)	0.0012<br>(0.0921)
Other multipolarized municipality	0.0703***<br>(0.0191)	0.068***<br>(0.0201)
Isolated municipality outside a hub influence	0.0449*<br>(0.0213)	0.0254<br>(0.0224)
Missing or inconsistent	-0.003<br>(0.03)	0.0049<br>(0.0317)
Past BZD use		
BZD use in the last quarter of the year preceding the WA	0.9331***<br>(0.0183)	0.9431***<br>(0.02)
BZD use in the third quarter of the year preceding the WA	0.3848***<br>(0.0147)	0.4385***<br>(0.0157)
BZD use in the second quarter of the year preceding the WA	0.0887***<br>(0.0139)	0.0931***<br>(0.0146)
BZD use in the first quarter of the year preceding the WA	0.1383***<br>(0.0131)	0.1567***<br>(0.0136)
BZD overuse in the previous year	1.4797***<br>(0.0126)	1.5606***<br>(0.0127)
Health		
Cancers	0.0344<br>(0.0189)	0.0328<br>(0.0195)
Cardioneurovascular diseases	0.1174***<br>(0.017)	0.1169***<br>(0.0173)
Vascular risk treatments (excluding	0.0468***	0.055***

pathologies)	(0.0108)	(0.0112)
Neurological or degenerative diseases	0.1748***<br>(0.0229)	0.1807***<br>(0.0232)
Psychiatric illnesses	0.4606***<br>(0.0137)	0.4429***<br>(0.0139)
Chronic end-stage renal disease	-0.0096<br>(0.0833)	0.045<br>(0.0851)
Chronic respiratory diseases	0.1246***<br>(0.0144)	0.1248***<br>(0.0148)
Other long-term conditions	0.0549**<br>(0.0212)	0.0557*<br>(0.0217)
Diabetes	0.1034***<br>(0.0166)	0.1277***<br>(0.017)
Diseases of the liver or pancreas	0.0899**<br>(0.0281)	0.0744**<br>(0.0284)
Maternity (with or without pathologies)	-0.0403<br>(0.0305)	-0.0472<br>(0.0339)
Addictive disorders	0.0408<br>(0.0248)	0.0417<br>(0.0246)
Antidepressant, lithium, Depakote, and Depamide treatments (excluding pathologies)	0.2753***<br>(0.0115)	0.2715***<br>(0.0118)
Neuroleptic treatments (excluding pathologies)	0.2995***<br>(0.0287)	0.2728***<br>(0.0287)
Prescriber position		
GP	Ref.	Ref.
Another specialist	-0.1202***<br>(0.0258)	-0.1519***<br>(0.0278)
Missing information	-0.5972***<br>(0.0149)	-0.6308***<br>(0.0162)
Multiple	0.2204*<br>(0.0962)	0.2192*<br>(0.0962)
Non-physician	0.0284<br>(0.0613)	0.0245<br>(0.0635)
Psychiatrist	0.1791***<br>(0.0156)	0.1546***<br>(0.0157)
Sex and age of the prescriber		
Missing information	0.0961***<br>(0.019)	0.0971***<br>(0.0198)
Male prescriber	0.0219*<br>(0.0109)	0.0263*<br>(0.0113)
Age < 40	Ref.	Ref.
Age 40-59	-0.0123<br>(0.0162)	-0.0094<br>(0.017)
Age > 59	0.0091<br>(0.0173)	0.007<br>(0.0181)
ρ (correlation coefficient of error terms)	0.2507***<br>(0.0298)	0.2437***<br>(0.0331)

Source: SNDS.
Field: People with WA in 2016 in France and randomly selected people without WA, using at least once a BZD after the WA date (N = 224,371).
*Note: * $p < 0.05$, ** $p < 0.01$, *** $p < 0.001$.*

Other health control variable

Table N: Results of the selection equation (1) with other health control variables

	**Coefficients (SE)**
WA	0.3285***<br>(0.0034)
Demographic	
Age in 2016	0.0087***<br>(0.0001)
Sex (ref. = male)	0.1704***<br>(0.0031)
Insurance	
CMU-C	0.0832***<br>(0.0046)
ACS	0.044***<br>(0.0076)
AAH	0.1913***<br>(0.0091)
Typology of the municipality	
Large hub (10,000 or more jobs)	Ref.
Outskirts of a large hub	-0.0034<br>(0.004)
Multipolarized municipality of large urban areas	-0.0051<br>(0.007)
Middle hub (5,000 to less than 10,000 jobs)	-0.0198*<br>(0.0092)
Outskirts of a middle hub	-0.0083<br>(0.0214)
Small hub (from 1,500 to less than 5,000 jobs)	0.0093<br>(0.0085)
Outskirts of a small hub	-0.0064<br>(0.0321)
Other multipolarized municipality	-0.0035<br>(0.0074)
Isolated municipality outside a hub influence	-0.0152<br>(0.0084)
Missing or inconsistent	-0.173***<br>(0.0107)
Past BZD use	
BZD use in the last quarter of the year preceding the WA	1.1381***<br>(0.0058)
BZD use in the third quarter of the year preceding the WA	0.7568***<br>(0.0061)
BZD use in the second quarter of the year preceding the WA	0.6211***<br>(0.0062)
BZD use in the first quarter of the year preceding the WA	0.6223***<br>(0.0061)
Health	
Decile of health expenditure in 2015	0.0411***<br>(0.0006)

Source: SNDS.
Field: People with WA in 2016 in France and randomly selected people without WA (N = 1,458,969).
*Note: * $p < 0.05$, ** $p < 0.01$, *** $p < 0.001$.*

Table O: Results of the outcome equation (2) with other health control variables

	Coefficients (SE)
WA	0.1074***<br>(0.0097)
Demographic	
Age in 2016	0.006***<br>(0.0003)
Sex (ref. = male)	-0.0493***<br>(0.008)
Insurance	
CMU-C	0.1329***<br>(0.0103)
ACS	0.0825***<br>(0.0156)
AAH	0.3501***<br>(0.0149)
Typology of the municipality	
Large hub (10,000 or more jobs)	Ref.
Outskirts of a large hub	0.0199*<br>(0.0098)
Multipolarized municipality of large urban areas	0.0448**<br>(0.017)
Middle hub (5,000 to less than 10,000 jobs)	0.0425*<br>(0.0216)
Outskirts of a middle hub	0.0692<br>(0.0517)
Small hub (from 1,500 to less than 5,000 jobs)	0.0855***<br>(0.0195)
Outskirts of a small hub	0.0419<br>(0.0788)
Other multipolarized municipality	0.0794***<br>(0.0174)
Isolated municipality outside a hub influence	0.0456*<br>(0.0196)
Missing or inconsistent	0.0145<br>(0.0272)
Past BZD use	
BZD use in the last quarter of the year preceding the WA	0.9962***<br>(0.0149)
BZD use in the third quarter of the year preceding the WA	0.3613***<br>(0.0133)
BZD use in the second quarter of the year preceding the WA	0.1506***<br>(0.0128)
BZD use in the first quarter of the year preceding the WA	0.2396***<br>(0.012)
BZD overuse in the previous year	1.3484***<br>(0.013)
Health	
Decile of health expenditure in 2015	0.0425***<br>(0.0016)
Prescriber position	
GP	Ref.
Another specialist	-0.158***<br>(0.0234)
Missing information	-0.583***<br>(0.0136)
Multiple	0.2837**<br>(0.0931)
Non-physician	0.1127*<br>(0.0567)
Psychiatrist	0.2676***<br>(0.0147)
Sex and age of the prescriber	
Missing information	0.1293***<br>(0.0174)

Male prescriber	0.0136<br>(0.01)
Age < 40	Ref.
Age 40-59	-0.0205<br>(0.0149)
Age > 59	0.0102<br>(0.0159)
ρ (correlation coefficient of error terms)	0.3847***<br>(0.0257)

Source: SNDS.
Field: People with WA in 2016 in France and randomly selected people without WA, using at least once a BZD after the WA date (N = 224,371).
*Note: * $p < 0.05$, ** $p < 0.01$, *** $p < 0.001$.*

Heterogeneity analysis results

Table P: Coefficients (SE) of the selection equation (1) by sex

	Men	Women
WA	0.2933***<br>(0.0052)	0.3669***<br>(0.0044)
Demographic		
Age in 2016	0.0104***<br>(0.0002)	0.0094***<br>(0.0002)
Insurance		
CMU-C	0.1194***<br>(0.0077)	0.1026***<br>(0.0058)
ACS	0.0541***<br>(0.0127)	0.0333***<br>(0.0095)
AAH	0.1214***<br>(0.0145)	0.0404**<br>(0.0134)
Typology of the municipality		
Large hub (10,000 or more jobs)	Ref.	Ref.
Outskirts of a large hub	-0.0242***<br>(0.0065)	0.0065<br>(0.0051)
Multipolarized municipality of large urban areas	-0.0207<br>(0.0114)	0.0069<br>(0.0089)
Middle hub (5,000 to less than 10,000 jobs)	-0.0261<br>(0.015)	-0.0235*<br>(0.0117)
Outskirts of a middle hub	0.012<br>(0.0343)	-0.02<br>(0.0273)
Small hub (from 1,500 to less than 5,000 jobs)	-0.0192<br>(0.014)	0.0194<br>(0.0108)
Outskirts of a small hub	0.0557<br>(0.0503)	-0.051<br>(0.0418)
Other multipolarized municipality	-0.0246*<br>(0.012)	0.0045<br>(0.0093)
Isolated municipality outside a hub influence	-0.0242<br>(0.0138)	-0.022*<br>(0.0106)
Missing or inconsistent	-0.2033***<br>(0.0171)	-0.1613***<br>(0.0138)
Past BZD use		
BZD use in the last quarter of the year preceding the WA	1.2186***<br>(0.01)	1.0727***<br>(0.0072)
BZD use in the third quarter of the year preceding the WA	0.7691***<br>(0.0106)	0.7212***<br>(0.0075)
BZD use in the second quarter of the year preceding the WA	0.6207***<br>(0.0109)	0.5827***<br>(0.0077)
BZD use in the first quarter of the year preceding the WA	0.609***<br>(0.0107)	0.5768***<br>(0.0075)
Health		
Cancers	0.0236<br>(0.0169)	0.0368***<br>(0.011)
Cardioneurovascular diseases	0.019	0.0157

	(0.0115)	(0.014)
Vascular risk treatments (excluding pathologies)	0.0263** (0.0081)	0.0198** (0.0065)
Inflammatory or rare diseases or HIV or AIDS	0.0829*** (0.0169)	0.0825*** (0.0132)
Neurological or degenerative diseases	0.1598*** (0.0193)	0.1091*** (0.0173)
Psychiatric illnesses	0.4001*** (0.0137)	0.4006*** (0.011)
Chronic end-stage renal disease	0.0712 (0.0602)	-0.0149 (0.064)
Chronic respiratory diseases	0.0807*** (0.0119)	0.1157*** (0.0091)
Other long-term conditions	0.0529** (0.0178)	0.0588*** (0.014)
Diabetes	-0.052*** (0.0117)	-0.0624*** (0.0111)
Diseases of the liver or pancreas	0.0823*** (0.0216)	0.0332 (0.0235)
Maternity (with or without pathologies)	NA	-0.0287*** (0.0082)
Addictive disorders	0.0972*** (0.024)	0.063* (0.0315)
Antidepressant, lithium, Depakote, and Depamide treatments (excluding pathologies)	0.3922*** (0.0126)	0.3701*** (0.0077)
Neuroleptic treatments (excluding pathologies)	0.211*** (0.0306)	0.186*** (0.0315)

Source: SNDS.
Field: Men (N = 634,786) and women (N = 824,183) among the study population.
*Note: * $p < 0.05$, ** $p < 0.01$, *** $p < 0.001$.*

Table Q: Coefficients (SE) of the outcome equation (2) by sex

	Men	Women
WA	0.0986*** (0.0159)	0.1181*** (0.013)
Demographic		
Age in 2016	0.0061*** (0.0007)	0.0051*** (0.0005)
Insurance		
CMU-C	0.185*** (0.0176)	0.1071*** (0.0134)
ACS	0.059* (0.0262)	0.0548** (0.0201)
AAH	0.2146*** (0.0244)	0.1786*** (0.0213)
Typology of the municipality		
Large hub (10,000 or more jobs)	Ref.	Ref.
Outskirts of a large hub	-0.0126 (0.0171)	0.0409** (0.0124)
Multipolarized municipality of large urban areas	-0.0293 (0.0296)	0.0985*** (0.0214)
Middle hub (5,000 to less than 10,000 jobs)	0.07 (0.0373)	0.0243 (0.0275)
Outskirts of a middle hub	0.0189 (0.0922)	0.1132 (0.0645)
Small hub (from 1,500 to less than 5,000 jobs)	0.0922** (0.0343)	0.0852*** (0.0245)
Outskirts of a small hub	0.0108 (0.1345)	0.0348 (0.1007)
Other multipolarized municipality	0.0685* (0.0307)	0.0863*** (0.0218)
Isolated municipality outside a hub influence	-0.0253 (0.0344)	0.0789** (0.0245)
Missing or inconsistent	0.0151	0.0235

	(0.0467)	(0.0349)
Past BZD use		
BZD use in the last quarter of the year preceding the WA	0.9346*** (0.0294)	0.9398*** (0.0201)
BZD use in the third quarter of the year preceding the WA	0.2863*** (0.0239)	0.3335*** (0.0173)
BZD use in the second quarter of the year preceding the WA	0.0916*** (0.0225)	0.1128*** (0.0165)
BZD use in the first quarter of the year preceding the WA	0.1501*** (0.0209)	0.1962*** (0.0153)
BZD overuse in the previous year	1.3453*** (0.0214)	1.3061*** (0.0161)
Health		
Cancers	0.0564 (0.0341)	0.0517* (0.0211)
Cardioneurovascular diseases	0.1256*** (0.0224)	0.1531*** (0.0246)
Vascular risk treatments (excluding pathologies)	0.001 (0.0176)	0.0798*** (0.0126)
Neurological or degenerative diseases	0.1603*** (0.0341)	0.1959*** (0.0298)
Psychiatric illnesses	0.4628*** (0.0226)	0.503*** (0.0167)
Chronic end-stage renal disease	-0.0401 (0.1167)	-0.0396 (0.1106)
Chronic respiratory diseases	0.1423*** (0.0237)	0.1401*** (0.0171)
Other long-term conditions	0.0619 (0.0345)	0.0343 (0.0256)
Diabetes	0.0728** (0.0244)	0.1212*** (0.0212)
Diseases of the liver or pancreas	0.1509*** (0.0371)	0.0989* (0.0414)
Maternity (with or without pathologies)	NA	-0.0034 (0.0268)
Addictive disorders	0.0271 (0.0333)	0.0954* (0.0396)
Antidepressant, lithium, Depakote, and Depamide treatments (excluding pathologies)	0.3006*** (0.0205)	0.2964*** (0.0132)
Neuroleptic treatments (excluding pathologies)	0.3041*** (0.042)	0.3225*** (0.04)
Prescriber position		
GP	Ref.	Ref.
Another specialist	-0.1419*** (0.0391)	-0.1254*** (0.0302)
Missing information	-0.568*** (0.0217)	-0.5605*** (0.0178)
Multiple	0.3195 (0.1677)	0.1412 (0.1156)
Non-physician	0.1045 (0.0885)	0.042 (0.0786)
Psychiatrist	0.1552*** (0.0254)	0.2129*** (0.0193)
Sex and age of the prescriber		
Missing information	0.0625* (0.0305)	0.1044*** (0.0223)
Male prescriber	-0.0112 (0.0185)	0.0281* (0.0123)
Age < 40	Ref.	Ref.
Age 40-59	-0.0432 (0.0267)	-0.0054 (0.0186)
Age > 59	0.0113 (0.0282)	0.0144 (0.02)
ρ (correlation coefficient of error terms)	0.2427*** (0.041)	0.316*** (0.0363)

Source: SNDS.

Field: Men (N = 79,129) and women (N = 145,242) using BZDs at least once after the WA date in the study population.
Note: * $p < 0.05$, ** $p < 0.01$, *** $p < 0.001$.
